# A study on the factors influencing old age identity among the Chinese elderly

**DOI:** 10.3389/fpubh.2022.1027678

**Published:** 2023-01-06

**Authors:** Xiao Yu, Qihui Wang

**Affiliations:** ^1^Northeast Asian Research Center, Jilin University, Changchun, China; ^2^Northeast Asian Studies College, Jilin University, Changchun, China

**Keywords:** old age identity, age values, intergenerational support, respect for the elderly, social identity

## Abstract

**Background:**

Old age identity is the self-perception of when old age begins, reflects public age values and acceptance of aging. Not all elderly people aged 60 and above identify with their old age status. The aging society in the traditional sense may not be “aging”. Therefore, redefining old age is crucial. Different from Western countries, China is a country with a long-standing culture of respect for the elderly and filial piety norms, and the influence of the cultural background on the old age identity is worthy of attention.

**Data and method:**

Data were drawn from the 2014 China Longitudinal Aging Social Survey (CLASS). A total of 7400 participants answered questions about old age identity. Based on old age identity, we obtained two other types of indicators of perceived old age: elderly group identity and aging degree. For the continuous variable old age identity and aging degree, an OLS linear regression model was established. A binary logistic regression model was established for the binary variable elderly group identity.

**Result:**

The average old age identity of Chinese people aged 60 and above is 70 years. Health status, psychological status, behavior, socioeconomic status, and some demographic characteristics significantly affect the old age identity of the Chinese elderly.

**Conclusion:**

The traditional Chinese cultural background of respect for the elderly and the norms of filial piety have an impact on the old age identity of the Chinese elderly through family intergenerational support. The various forms of support provided by children to the elderly can help them achieve a positive age identity—enter old age later, raise the likelihood of identification with non-elderly groups, and reduce aging degree. The number of children of the elderly, the children's economic conditions and care support for grandchildren have negative effects. A positive old age identity can help the elderly improve their self-esteem.

## Introduction

China—along with most countries in the world —are facing severe aging problems. The increase in the elderly population will not only increase the support burden of young people, but also lead to major challenges for employment, the social security system, health care system, etc (1–3). Statistics from the United Nations show that in 2017, the world's elderly population aged 60 and above accounted for 13% of the total population, and more than 100 countries and regions have entered an aging society; China's elderly population aged 60 and above is about 226 million, equivalent to nearly a quarter of the world's elderly population, accounting for 16% of China's population (4). However, this is only based on the statistics of the elderly at the age of 60 as the benchmark for old age. In the past 30 years, the average life expectancy in China has increased from 67.9 to 76.5 years (5). As life expectancy increases, individuals also change their perceptions of when they enter old age (6, 7) and may reassess their own perceptions of aging (8). Older people do not necessarily consider themselves old (9, 10), and chronological age is no longer the only useful indicator for defining old age (11). There are also studies suggesting that 15 years of expected remaining lifespan should be used as a new standard for the definition of old age (12). Advances in longevity, healthy aging, and technological innovation have made today's elderly become a generation that actively contributes to society and the family, so it may be meaningless to define old age in a static, homogenous way (13).

According to the 2014 China Longitudinal Aging Social Survey, the average chronological age of 7,399 elderly people aged 60 and above is about 69 years, but the average self-perception of aging is about 70 years. China and many other countries still define the elderly as 60 years old and above, but based on the old age identity (the self-perception of when old age begins), China and other countries in the world still have room to maneuver in response to the challenges of aging. Not all Chinese elderly people aged 60 and above identify with their old age status. Therefore, the practical outcome of self-identity of old age may be that the actual scale of the elderly would be reduced, and the aging society in the traditional sense may not be “aging.” In addition, China uses chronological age as the age reference for mandatory retirement. The Chinese government stipulates that the legal retirement age is 60 for men, 55 for women in white-collar jobs, and 50 for women in blue-collar jobs. Although there is no consensus on when a person ages, 60 or 65 years is the most commonly used eligibility age for pension plans, and therefore people associate retirement age with old age (14). In order to cope with the many challenges brought by an aging society, postponing the retirement age can ease the pressure on pensions and increase labor supply. However, based on the old age identity, postponing the retirement age may be more in line with the psychological expectations of the old age identities over 60, and bring certain negative psychological impacts to the old age identities below 60. Therefore, the policy significance of old age identity is reflected in the fact that it may affect public response and acceptance of relevant policies such as the gradual extension of retirement age. When adjusting the retirement age, referring to old age identity will help improve the flexibility of the policy.

The old age identity of the Chinese elderly can reflect the age values and the acceptance of aging of the contemporary Chinese public. Paying attention to the old age identity of the Chinese elderly has great practical and policy significance for actively responding to the aging society. We believe that redefining old age is crucial. Combined with the existing research on the influencing factors of old age identity, we found that there are three shortcomings: the construction of old age identity is based on a single index, the influencing factors rarely involve cultural background, and the significance of old age identity to the elderly is not highlighted. Therefore, this paper addresses the following questions:

RQ1: What is the characteristic of the old age identity of the Chinese elderly?

RQ2: What factors influence the old age identity of the Chinese elderly, especially whether cultural background factors have a significant impact?

RQ3: What is the significance of old age identy for the elderly, why do some elderly people tend to enter old age later?

Answers to these questions will help identify differences in the old age identities of various types of elderly people, thereby providing greater flexibility for the formulation of public policies involving retirement and old-age security. This study takes China as an example, based on demographic correlates, socioeconomic status, health status, psychological status, behavior and major events, combined with the traditional Chinese cultural background of respect for the elderly and filial piety norms, to focus on the influence of family factors, especially family intergenerational support, on the old age identity of the Chinese elderly. Based on the social identity theory of social psychology (15–18), we empirically analyzed the significance of old age identity for the elderly, that is, whether positive old age identity helps the elderly improve self-esteem.

## Literature review

Old age identity is the self-perception of when old age begins. Kaufman and Elder proposed five dimensions of age identity, including subjective age (the age you feel like most of the time), other age (the age other people think you are), desired age (the age you want to be), desired longevity (the age you hope to live to) and perceived old age (the age at which you become old) (8). Diehl et al. emphasized that age identity originated from the role identity theory of sociology and the social identity theory of social psychology in their seminal theoretical reflections on the awareness of aging (8, 15, 19, 20). Individuals explicitly and consciously report their age identities to ensure that they belong to the most appropriate identity, from among the diverse social role identities represented by the various age classes (21, 22). Age identity is the core of social identity (15), and individuals belong to certain social groups according to their age identities as group members. Therefore, consistent with Goffman's definition of identity and with overall sociological and social-psychological theory, age identity is primarily seen as a person's subjective sense of age based on their social experiences and identification with a particular age group (19, 23).

There is accumulating evidence that age identity relates to a wide array of physical and mental health outcomes, including mortality, functional status, morbidity and loneliness (24, 25). Age identity has a better effect than chronological age in predicting cognitive aging patterns (26). In recent years, the influence of age identity on workplace performance has received much attention in the field of psychology (27–29). The fact that subjective age identity influences the ability as well as the motivation to act or perform (30), makes it a vital personal characteristic to explore in the domain of economic behaviors (31).

As age identity shows amazing predictive power in different domains, more studies have begun to focus on the factors influencing age identity. Most studies on the factors influencing age identity are based on four types of potential related factors proposed by Barak & Stern: (a) biological and physiological correlates (i.e., objective health status or self-perceived health); (b) demographic correlates (i.e., gender, marital status, socio-economic status, etc.,); (c) socio-psychological correlates (i.e., life satisfaction, emotional health, morale etc.,); and (d) behavioral correlates (i.e., consumer behavior, leisure-time activities, etc.) (32). Health is an important factor influencing age identity (33, 34), and those with more positive evaluations of cognitive and physical function tend to delay the onset of old age (35). Chronic diseases were positively correlated with subjective age (36). Barak and Rahtz found that there were no significant differences in subjective age and the likelihood of feeling older between men and women (37). Recent research has further demonstrated that the subjective age of young and middle-aged adults is not related to gender (38). Participants who reported higher levels of financial stress were perceived as older than their actual age to a greater extent (39). Based on 2471 investigators aged 40–79, Bergland et al. found that factors such as older age, physical health, mental health and higher personal accomplishment can significantly predict perceived subjective younger age (40). Grandparents are critical to the shaping of personal aging experiences, and grandchild care behaviors significantly influence age identities of the elderly (41, 42).

Recent research provides empirical support for views of aging from a life-course perspective, emphasizing that childhood starvation experience predict older subjective age and that parental death is associated with lower old age identity (43). Barrett and Gumber examine the impact of aging body reminders on age identity, such as everyday body problems and body repairs (44). While most studies have considered age identity to be a relatively stable variable, Hughes and Touron offer a novel account of how daily life offers a variety of situational contexts and experiences that directly impact the age a person feels at a given moment (45). It has also been found that retirement intentions positively predicted subjective age (46), while personality traits with higher scores such as extroversion, openness, agreeableness and conscientiousness were associated with a younger subjective age (47). The closest study to this paper is Liu et al., who focused on examining the impact of social engagement behaviors on the old age identities of the Chinese elderly and found that political engagement behaviors of the elderly help delay the onset of old age (11).

Most studies on age identity focus on subjective age identity and old age identity. However, there are still three insufficiencies in the studies on the influencing factors of old age identity: First, it is difficult to explain the profound connotation of old age identity with a single research index. Most studies only conduct empirical analyses based on continuous variables measured by age identity, but ignore that age identity also reflects identity with a specific age group, furthermore, continuous variables derived from age identity measures can be combined with chronological age to perform mathematically more complex analyses (48). The second limitation is the lack of cultural background factors. Age identity is based on the understanding of individuals' cognitions and experiences in the sociocultural context (24). By contrasting American and German age identities, Westerhof and Barrett emphasized that gerontological theory and research should incorporate the cultural context (49). Third, the significance of old age identity for the elderly is not highlighted in the empirical analyses. Most studies on age identity ignore the process of self-perception and meaning formation (19), and deciding how respondents feel about a particular age remains a challenge (50). We therefore ask the question: do the elderly achieve self-enhancement through a positive age identity?

In view of the insufficiencies of existing studies, we advance the research on the influencing factors of old age identity from the following three aspects.

First, on the basis of old age identity, we combine the theoretical connotation of age identity and draw on the mathematical processing technology of Rubin and Berntsen to predict subjective age identity (the age you feel like most of the time) (48). Using the same mechanism, we can logically deduce the question of old age identity (the self-perception of when old age begins), and obtain two other types of indicators of perceived old age: elderly group identity and aging degree.

Second, we combine the traditional Chinese cultural background of respect for the elderly and incorporate family factors such as family intergenerational support into the analysis of the factors influencing the old age identity of the Chinese elderly. Age discrimination is a universal feature of European countries (51); a number of older people in the world may suffer from disrespect, and only a few can maintain the respect of younger generations through unusual achievements (52, 53). However, China is a society with a traditional culture of respect for the elderly (54), which is also reflected in other East Asian countries, such as Japan and South Korea (55, 56). China is a country with very close two-way support between parents and children. Family intergenerational support such as financial, emotional, and domestic support provided by children to the elderly is considered a behavior of respect for the elderly (54), which is also in line with Chinese traditional filial piety norms (57). The elderly often also provide care and support for their grandchildren.

Third, to provide a new perspective to the study of the factors influencing the old age identity of the elderly, based on the social identity theory of social psychology, this paper empirically analyzes the significance of old age identity to the elderly, that is, the way in which a positive old age identity can help the elderly improve their self-esteem. Social identity is defined as an individual's psychological process of obtaining emotional experience and sense of worth as a member of a specific social group (16). Social identity theory argues that through appropriate intergroup social comparison, positive social identity can be obtained, for example, enhancing self-esteem (15, 17, 18). To some extent, age identity may serve the purpose of compensation and self-enhancement (58–60). For the elderly, the importance of this self-enhancement is even greater than that of health and socioeconomic status in predicting age identity (49).

## Materials and methods

### Data

Data were drawn from the 2014 China Longitudinal Aging Social Survey (CLASS). CLASS is a nationwide and continuous large-scale social survey project, specifically implemented by the China Survey and Data Center of Renmin University of China. By regularly and systematically collecting the social and economic background data of the elderly in China, CLASS can grasp the various problems and challenges faced by the elderly in the aging process, evaluate the actual effects of various social policy measures in improving the quality of life of the elderly, and provide important theoretical and factual basis for solving the problem of aging in China. The first nationwide baseline survey was carried out in 2014, and an elderly person aged 60 and above was selected from the sampled households for interview, and a total of 11,511 resident questionnaires were completed. A total of 7,400 participants answered questions about old age identity. Of the 4,111 participants who did not answer questions about old age identity, 2,015 were unable to answer these questions due to lower cognitive functioning or poor understanding of the questions. Compared to those who completed the study, those who were excluded from the study were older, had lower levels of education and income, and a higher proportion were female residents of rural areas. According to the needs of the research, we retain a total of 6,788 samples of elderly people whose age was between 40 and 100 and had 1–5 children. After eliminating the missing values of the variables, the final sample size was 6,033.

### Measures

Based on four types of potential related factors proposed by Barak and Stern (32), and combined with the traditional Chinese cultural background of respect for the elderly, we built a predictive model of old age identity among the Chinese elderly, including seven dimensions: demographic correlates (age, gender, marital status, ethnicity, extent of solitude, residence), socioeconomic status (educational achievement, pension income, occupation type), health status (self-assessed health, chronic diseases), psychological status (life satisfaction, emotional status), behavior (economic behavior, political behavior, public welfare behavior), major events (major sad events, major happy events), and family factors (the number of children, children's economic conditions, care support for grandchildren, emotional support provided by the children, financial support provided by the children, domestic support provided by the children).

### Old age identity

Old age identity not only reflects the specific age identified by individuals, but also, combined with the chronological age (CA), can further reflect the differences in the aging degree of individuals and the identity with a particular age group. Compared with previous studies, we use three types of indicators to interpret and enrich the profound connotation of old age identity, which is conducive to the in-depth study of the factors influencing age identity. Details as follows: First, CLASS2014 asked the respondents “At what age do you think you are old?”. This is the respondents' self-perception of when old age begins, and we measure old age identity accordingly. Second, Rubin and Berntsen introduced three measures of subjective age identity, subjective age (SA); absolute difference between subjective age and chronological age (SA - CA); and relative difference between subjective age and chronological age (SA - CA) / CA (48). Drawing on Rubin and Berntsen's mathematical processing techniques for subjective age identity, we use two methods to measure the aging degree, absolute difference between chronological age and old age (CA - OA), and relative difference between chronological age and old age (CA - OA) / OA. For example, if old age identity is 60 years old and chronological age is 70 years old, aging degree is 10 years old or 16.7%; if old age identity is 65 years old and chronological age is 75 years old, aging degree is 10 years old or 15.4%. Third, based on the social identity theory, old age identity reflects identity with a particular age group. When a nominal elderly person over 60 years old answers the self-perception of when old age begins, it also represents their own judgment on the identity of an age group, that is, whether they belong to the elderly group or not. This is something that cannot be measured by the index system of Rubin and Berntsen, and is also the focus of this study. Therefore, elderly group identity can be expressed as two groups: the non-elderly group (OA > CA), and the elderly group (OA ≤ CA).

### Demographic correlates

Demographic correlates include age, gender, marital status, ethnicity, extent of solitude, and residence. Age is a continuous variable, and gender (male = 1, female = 0), marital status (married = 1, not married = 0), ethnicity (Han Chinese = 1, non-Han Chinese = 0), extent of solitude (live alone = 1, do not live alone = 0), residence (urban = 1, rural = 0) are set as categorical variables.

### Socioeconomic status

Socioeconomic status includes educational achievement, pension income, and occupation type. Occupation type is a categorical variable measured by respondents' most recent primary occupation (whether currently working or previously working), agricultural jobs (including farming, animal husbandry and fishing) = 0, or non-agricultural jobs = 1. Educational achievement and pension income are continuous variables. Educational achievement is measured by years of education (illiterate or never attended primary school = 0, primary school = 6, junior high school = 9, high school/technical secondary school = 12, junior college and above = 15). In China, an individual's pension type is related to his or her occupational background, including basic pension for urban employees, retirement pension for government agencies and institutions, social pension for urban residents and social pension for rural residents. CLASS2014 asked the respondents about the above pension income, the unit is 1,000RMB.

### Health and psychological status

Health status includes the categorical variables of self-assessed health and chronic diseases. Psychological status includes life satisfaction and emotional status. The measure of chronic disease was: 1 if there is a chronic disease, 0 otherwise. In this paper, referring to the practice of Bordone et al. (13), self-assessed health and life satisfaction were set as two-category variables. The measure of self-assessed health was: healthy (relatively healthy, very healthy) = 1, unhealthy (generally unhealthy, relatively unhealthy, and very unhealthy) = 0. The measure of life satisfaction was: satisfaction (relatively satisfied, very satisfied) = 1, dissatisfied (generally dissatisfied, relatively dissatisfied, very dissatisfied) = 0. CLASS2014 asked the elderly about their emotional status in the past week from the time of the survey, with questions such as “Did you feel lonely in the past week?”, “Did you feel sad in the past week?”, “Did you feel that you didn't want to eat in the past week?”, and “Did you sleep poorly in the past week?”, with the responses of often = 1, sometimes = 2, and never = 3. We added up the scores for the 4 questions about emotional status; the score range of emotional status was 4–12 points, the higher the score, the better the emotional status.

### Behavior

Behavioral correlates include economic behavior, public welfare behavior, and political behavior. Economic behavior is measured by questions such as “Are you currently engaged in a paid job?”, and scored as 1 if the answer is yes, and 0 otherwise. Political behavior is measured by questions such as “Have you participated in the voting of the local residents committee or village committee in the past 3 years?”, with a score of 1 if the answer is yes, 0 otherwise. The measure of public welfare behavior is participation in public welfare activities in the past 3 months from the time of the survey. Public welfare activities are divided into seven categories: community security patrols, caring for other elderly members, environmental sanitation protection, mediation of disputes, accompanying chat, volunteer services requiring professional skills, and helping to take care of children from other families, with a score of 0 if the respondent has attended, but not in the past 3 months, or has never attended, 1 otherwise.

### Major events

We considered two types of major events: One was major happy events, and the measurement standard was two major events of child or grandchild marriage, child or grandchild birth in the past 12 months since the time of the survey, with a score of 1 if the respondent experienced at least 1 event, 0 otherwise. The other was major sad events, and the measurement standard was based on nine major events of serious illness, natural disaster, death of spouse, death of child, death of other relatives and friends, financial loss, serious illness of family members, conflicts with relatives and friends, and accidents in the past 12 months since the time of the survey, with a score of 1 if the respondent experienced at least 1 event, 0 otherwise.

### Family factors

The traditional Chinese culture of respect for elders stems from Confucian teachings of filial piety; filial piety essentially directs offspring to recognize the care and aid received from their parents and, in return, to pay respect for their parents (61). In Chinese society, adult children attach great importance to filial piety, and the dominant value of filial piety stipulates that adult children assume the responsibility of caring for their aging parents (57). Combining the traditional Chinese cultural background of respect for the elderly and the norms of filial piety, this paper focuses on the influence of family intergenerational support on the old age identity of the Chinese elderly in relation to family factors. In addition to the three forms of support provided by children to their elders in line with filial piety, grandchild care support, number of children, and children's economic conditions are also included in family factors.

CLASS2014 asked about the financial support provided by the children to the elderly respondents in the past 12 months from the time of the survey, including money, food or gifts, converted into a range of monetary amounts for children to choose from, such as “not provided,” “1~199RMB,” “200~499RMB,” etc. We used the median of the range of monetary amounts to approximate the financial support provided to respondents by each child. CLASS2014 counts financial aid for up to 5 children, therefore, the number of children in the empirical sample of this paper is 1–5, and children's financial support is the average monthly amount of money provided by all the children of the respondents. Children's housework support was measured by questions such as “how often did this child help you with housework in the past 12 months?”, if the respondent answered “almost every day,” a score of 1 was given, otherwise it was 0, other options included “at least once a week,” “at least once a month,” “several times a year” and “hardly ever.” Children's emotional support was measured by questions such as “do you think this child does not care enough for you?”, if the respondents answered “never,” a score of 1 was given, otherwise it was 0, other options included “occasionally,” “sometimes” and “often.” CLASS2014 asked respondents how often they had looked after their grandchildren in the past 12 months since the time of the survey, with options including “from morning to night,” “for some time each day,” “at least once a week,” “several times a month,” “about once a month,” “rarely,” or “not.” If the respondent had at least one grandchild whose frequency of grandchild care is from morning to night or some time each day, the score for care support for grandchildren was 1, otherwise it was 0. The children's economic conditions was measured by questions such as “how do you think the child's financial condition is?”, if the elderly answered “relatively difficult” or “very difficult” for all their children's financial situations, the score was 1, otherwise it was 0, other options include “basically adequate,” “relatively well-off” and “very well-off.”

### Methods

In this study, an OLS linear regression model and a binary logistic regression model were used to empirically analyze the influencing factors of the three types of indicators of old age identity. For the continuous variable old age identity and aging degree, an OLS linear regression model was established, see model (1) and model (2). A binary logistic regression model was established for the binary variable elderly group identity, see model (3).


(1) $$\begin{array}{l} Y_{1}=\alpha +\beta_{1}X_{1}+\cdots +\beta_{k}X_{k}+\varepsilon \end{array}$$

(2) $$\begin{array}{l} Y_{2}=\alpha +\beta_{1}X_{1}+\cdots +\beta_{k}X_{k}+\varepsilon \end{array}$$

(3) $$\begin{array}{l} ln\left[P/\left(1-p\right)\right]=\alpha +\beta_{1}X_{1}+\cdots +\beta_{k}X_{k}+\varepsilon \end{array}$$

Among them, *Y*_1_ represents old age identity, *P* is the probability that the elderly identify themselves as belonging to the non-elderly group, *X* is the independent variable, α is the constant term, β is the coefficient to be estimated, and ε is the error term. For the estimation of the direction of the coefficient to be consistent, this paper sets *Y*_2_ as the opposite number of the aging degree. Table 1 shows the descriptive statistics of the variables.

**Table 1:** Descriptive statistics of the variables.

**Continuous variables**	**Average**	**Mean**	**Minimum**	**Maximum**
Old age identity	70.05	9.77	40	100
Aging degree (absolute difference)	−1.04	10.44	−40	36
Aging degree (relative difference)	0	0.15	−0.4	0.9
Age	70.31	8.10	60	113
Educational achievement	5.63	4.72	0	15
Pension income	1.17	1.44	0	10.23
Emotional status	10.24	1.83	1	12
The number of children	2.76	1.20	1	5
Financial support provided by the children	0.31	0.43	0	5
**Categorial variables**	**Percentages (%)**	
	0	1
Elderly group identity	53.63	46.37
Gender	52.04	47.96
Marital status	35.21	64.79
Ethnicity	7.54	92.46
Extent of solitude	86	14
Residence	40	60
Occupation type	48.28	51.72
Self-assessed health	57.58	42.42
Chronic diseases	25.32	74.68
Life satisfaction	24.75	75.25
Economic behavior	81	19
Political behavior	54.16	45.84
Public welfare behavior	79.86	20.14
Major happy events	89.82	10.18
Major sad events	71.23	28.77
Children's economic conditions	88	12
Care support for grandchildren	71.55	28.45
Emotional support provided by the children	18.58	81.42
Domestic support provided by the children	67.90	32.10

## Results

Table 2 shows the old age identity of the Chinese elderly based on CLASS2014. Excluding the sample of respondents whose chronological age was missing (only 1), 7,399 respondents aged 60 and above participated in the survey on old age identity. The average chronological age of the participants was about 69.02 years, and the average old age identity was about 70.04 years. It can be seen that the average chronological age of the Chinese elderly is less than the average old age identity. However, through the elderly group identity, we found that the elderly who identified themselves as the elderly group (CA ≥ OA) accounted for 54%, the average chronological age was about 70.83 years, and the average old age identity was about 64.31 years; the elderly who belonged to the non-elderly groups (CA < OA) accounted for 46%, the average chronological age was about 66.92 years, and the average old age identity was about 76.70 years. We believe that old age identity among Chinese elderly does reflect identity with a specific age group.

**Table 2:** The old age identity of the Chinese elderly based on CLASS2014.

**Variable**		**Sample**	**Average chronological age**	**Average old age identity**	**T-values**
-		7,399	69.02	70.04	-
Gender	Male	3,966	68.91	70.13	−0.83
	Female	3,374	69.11	69.98	
Residence	Urban	4,768	69.41	71.23	−13.81^***^
	Rural	2,623	68.30	67.92	
Self-assessed health	Health	3330	68.37	71.25	−8.62^***^
	Unhealth	4,056	69.55	69.08	
Educational achievement	Primary school and below	4,243	69.64	68.88	−11.75^***^
	Junior high school and above	3,148	68.18	71.65	
Public welfare behavior	Participate	1,616	68.11	70.51	−2.06^**^
	Not participate	5767	69.27	69.92	

^*^p < 0.1,

** *p* < 0.05,

*** *p* < 0.01.

Due to space limitations, we select a few of the factors to compare the old age identity of the various categories of the Chinese elderly. We found that the average old age identity of male and female Chinese elderly members was almost the same, 70 years. However, the average old age identity of the Chinese elderly living in cities was 71.23 years, which is significantly higher than that of the Chinese elderly living in rural areas, which is 67.92 years. It was found that elderly people with better self-assessed health status have a higher average old age identity. The average old age identity of the elderly who self-assessed their health as unhealthy was 69.08 years, and the average old age identity of the elderly who self-assessed their health as healthy was 71.25 years. The higher the educational achievement of the elderly, the higher the average old age identity. The average old age identity of the elderly with an education level of primary school and below was 68.88 years, the average old age identity of the elderly with an education level of junior high school was 71.07 years, and that of the elderly with an education level of junior high school and above was 71.65 years. The average old age identity of the elderly who participated in public welfare behaviors was 70.51 years, and that of the elderly who did not, was 69.92 years. According to the T-values and significance results that the mean *T*-test report, we found that, except for gender, there are significant differences in old age identity among different categories of the Chinese elderly, however, these differences may be influenced by other variables. Therefore, it is necessary to establish a regression model to exclude the influence of other related variables, and to examine whether the independent effects of each variable on the old age identity of the Chinese elderly are significant.

Table 3 presents the empirical results of the factors influencing old age identity among the Chinese elderly.

**Table 3:** Empirical results of the factors influencing old age identity among the Chinese elderly.

**Variable**	**Old age identity**		**Elderly group identity**		**Aging degree (relative difference)**	
**Demographic correlates**						
Age	0.387^***^	(0.022)	−0.098^***^	(0.006)	−0.009^***^	(0.000)
Gender	−0.303	(0.248)	−0.001	(0.064)	−0.005	(0.004)
Marital status	−0.021	(0.331)	−0.078	(0.086)	−0.002	(0.005)
Ethnicity	−0.016	(0.525)	−0.021	(0.133)	0.002	(0.008)
Extent of solitude	0.424	(0.450)	0.002	(0.111)	0.003	(0.007)
Residence	0.114	(0.329)	0.091	(0.085)	0.002	(0.005)
**Socioeconomic status**						
Educational achievement	0.095^***^	(0.034)	0.032^***^	(0.009)	0.002^***^	(0.000)
Pension income	0.463^***^	(0.122)	0.112^***^	(0.031)	0.007^***^	(0.002)
Occupation type	1.453^***^	(0.355)	0.282^***^	(0.090)	0.021^***^	(0.005)
**Health status**						
Self-assessed health	1.296^***^	(0.248)	0.327^***^	(0.064)	0.018^***^	(0.004)
Chronic diseases	−1.250^***^	(0.288)	−0.273^***^	(0.071)	−0.014^***^	(0.004)
**Psychological status**						
Life satisfaction	1.006^***^	(0.288)	0.262^***^	(0.077)	0.015^***^	(0.004)
Emotional status	0.402^***^	(0.069)	0.086^***^	(0.018)	0.005^***^	(0.001)
**Behavior**						
Economic behavior	−0.172	(0.313)	−0.001	(0.082)	−0.002	(0.005)
Political behavior	0.499^**^	(0.234)	0.155^**^	(0.061)	0.007^**^	(0.003)
Public welfare behavior	0.675^**^	(0.282)	0.173^**^	(0.072)	0.009^**^	(0.004)
**Major events**						
Major sad events	−0.079	(0.261)	−0.062	(0.068)	−0.002	(0.004)
Major happy events	−0.354	(0.387)	−0.035	(0.104)	−0.004	(0.006)
**Family factors**						
The number of children	−0.416^***^	(0.123)	−0.114^***^	(0.031)	−0.006^***^	(0.002)
Children's economic conditions	−1.072^***^	(0.406)	−0.281^**^	(0.111)	−0.015^***^	(0.006)
Care support for grandchildren	−0.701^***^	(0.261)	−0.146^**^	(0.068)	−0.011^***^	(0.004)
Emotional support provided by the children	0.715^**^	(0.298)	0.127^*^	(0.077)	0.011^**^	(0.004)
Financial support provided by the children	0.651^**^	(0.298)	0.128^*^	(0.072)	0.008^**^	(0.004)
Domestic support provided by the children	0.530^*^	(0.277)	0.137^*^	(0.070)	0.007^*^	(0.004)
Constant term	36.932^***^	(1.723)	5.305^***^	(0.457)	0.516^***^	(0.025)
R^2^/ Pseuto R^2^	0.164		0.133		0.250	
Sample	6,033		5,553		6,033	

* *p* < 0.1,

** *p* < 0.05,

*** *p* < 0.01, values in parentheses are robust standard errors clustered to the individual level. The empirical results of the aging degree measured by absolute difference are consistent with the old age identity, hence they are not presented here, however, they are available upon request. Considering that the elderly with the same old age identity and chronological age may be in the transition stage between the elderly group and the non-elderly group, in order to highlight the difference in the influence of various factors on the identity of the elderly group, we excluded the elderly with the same old age identity and chronological age. There are a total of 480 samples in this part.

First, among the demographic correlates, only age has a significant impact on the three indicators of old age identity. Increasing age causes the elderly to enter old age later, raise the likelihood of identification with the elderly group, and exacerbate aging degree. Gender, marital status, ethnicity, extent of solitude, and residence have no significant effects on the three indicators of old age identity. Studies have also shown that, in addition to the effect of chronological age, demographic correlates play a secondary role at best in the analysis of the impact of age identity (48, 62, 63). Socioeconomic status, educational achievement, pension income and occupation type, all have significant effects on the three indicators of old age identity. Higher years of schooling and higher pension income cause the elderly to enter old age later, raise the likelihood of identification with non-elderly groups, and reduce aging degree. Compared with agricultural work, non-agricultural work causes the elderly to enter old age later, raise the likelihood of identification with non-elderly groups, and reduce aging degree. This is consistent with earlier research conclusions that socioeconomic status can reflect different social experiences of older adults, while age identity is primarily viewed as a person's subjective sense of age based on their diverse social experiences (19). Socioeconomic status affects age identity; those with poorer socioeconomic status have older identities (64). Recent research has also shown that there is heterogeneity in how people feel about old age when it comes to education, for example, highly educated respondents were less likely to associate old age with loneliness and boredom than respondents with lower education levels (13).

Second, health and psychological status have a significant impact on the three indicators of old age identity. Better self-assessed health, no chronic disease, higher life satisfaction status, and better emotional status cause the elderly to enter old age later, raise the likelihood of identification with non-elderly groups, and reduce aging degree. Among the behaviors, political and public welfare behavior have a significant impact on the three indicators of old age identity. Participating in political elections and public welfare activities cause the elderly to enter old age later, raise the likelihood of identification with non-elderly groups, and reduce aging degree. Although work plays a central role in the formation of self-perception (14), economic behavior has no significant effect on the old age identity of the Chinese elderly, which is consistent with the findings of Liu et al. (11). In addition, neither of the two major types of events (happy or sad) had a significant effect on old age identity. Since CLASS2014 counts the major events experienced by the elderly in the past year, we speculate that the impact of major events experienced by the elderly may be time-sensitive, and may affect old age identity by acting on the psychology of the elderly in a short period of time.

Finally, in the context of the traditional Chinese culture of respect for the elderly, there are positive and negative effects of family-related factors on the three types of indicators of old age identity among the Chinese elderly. The financial, emotional, and domestic support provided by children to the elderly have positive effects. Children providing substantial financial support to the elderly, caring about them often, and helping them with housework often, enable them to enter old age later, raise the likelihood of identification with non-elderly groups, and reduce aging degree. Studies have shown that more than 50% of the elderly aged 64–74 years believe that the decline in physical health and loneliness are the main reasons for their aging (13). These intergenerational support behaviors of respect for the elderly and filial piety may greatly improve the material and spiritual life of the elderly, help improve their physical health, and reduce their loneliness, so that they may harbor a more positive old age identity. The number of children of the elderly, the children's economic conditions and care support for grandchildren have negative effects. Larger numbers of children, financially disadvantaged children, and often caring for grandchildren cause the elderly to enter old age earlier, raise the likelihood of identification with elderly groups, and exacerbate aging degree. This paper argues that the elderly may devote more energy to raising a larger number of children, the difficult economic conditions of all children may bring psychological burdens to the elderly, and taking care of grandchildren often may also decline the physical health of the elderly. These effects may make it difficult for the elderly to demonstrate a more positive old age identity.

To test whether the elderly achieve self-enhancement through positive age identity, in this paper, a two-category proxy variable of self-esteem of the elderly is set up, and a logistic model is constructed. Based on the social identity theory of social psychology (15–18), we empirically analyzed the significance of old age identity for the elderly, that is, whether positive old age identity helps the elderly improve self-esteem. Up to now, the most widely used self-esteem scale (Rosenberg Self-Esteem Scale, RSE) is an assessment of an individual's overall feelings of self-worth and self-acceptance (65). Since CLASS2014 did not set up such self-esteem scales, this study only establishes the two-category proxy variable of the elderly's self-esteem through the question set by CLASS2014 namely: “I think I am still a useful person to society, is this statement consistent with the actual current situation of the elderly?”. Set the answers “completely inconsistent,” “relatively inconsistent,” “general” to 0, and “more consistent” and “completely consistent” to 1. This question of CLASS2014 is more consistent with the evaluation indicators in RSE: “I feel that I am a valuable person” and “I have a positive attitude toward myself,” which reflects the affirmation of the self-worth of the elderly. In addition, to be more precise, this question of CLASS2014 reflects a specific self-esteem (for a specific self-image, as opposed to the overall self-image) and state self-esteem (self-evaluation for the moment, as opposed to self-evaluation over a longer period of time) (66). Some studies have also pointed out that group classification under social identity theory is more likely to have an impact on state self-esteem that reflects current self-evaluation (67). Therefore, the setting of the proxy variable of elderly self-esteem in this paper has a certain representativeness.

Table 4 reports the self-enhancement effect of old age identity among the Chinese elderly. The empirical results show that the group identification of the elderly significantly affects their self-esteem. In order to improve self-esteem, the elderly tend to identify themselves as belonging to the non-elderly group. This is consistent with the previous theoretical analysis. Through the social comparison between the non-elderly group and the elderly group, a positive social identity can be obtained, so as to achieve the purpose of self-enhancement. In addition, we further examined the effects of old age identity and aging degree on the self-esteem of the elderly. We found that in order to improve self-esteem, the elderly tend to enter old age later and have a lower aging degree.

**Table 4:** The self-enhancement effect of old age identity among the Chinese elderly.

	**(1)**	**(2)**	**(3)**	**(4)**	**(5)**	**(6)**	**(7)**	**(8)**
Elderly group identity	0.515^***^ (0.059)	0.543^***^ (0.086)						
Elderly group identity^*^Gender		−0.050 (0.113)						
Old age identity			0.030^***^ (0.003)	0.037^***^ (0.005)				
Old age identity^*^Gender				−0.013^**^ (0.006)				
Aging degree (absolute difference)					0.030^***^ (0.003)	0.037^***^ (0.005)		
Aging degree (absolute difference)^*^Gender						−0.012^**^ (0.006)		
Aging degree (relative difference)							2.064^***^ (0.233)	2.553^***^ (0.342)
Aging degree (relative difference)^*^Gender								−0.838^**^ (0.425)
Control variables	control	control	control	control	control	control	control	control
R^2^/Pseudo R^2^	0.092	0.092	0.094	0.095	0.094	0.095	0.093	0.094
Sample	5,917	5,917	5,917	5,917	5,917	5,917	5,917	5,917

* *p* < 0.1,

** *p* < 0.05,

*** *p* < 0.01, values in parentheses are robust standard errors clustered to the individual level. Control variables include demographic correlates, socioeconomic status, health status, psychological status, behavior, major events, and family factors.

However, male and females differently perceive things (68, 69). Recent research shows that gender moderated the relationships between social interaction activities and self-esteem, females reported higher levels of engagement in social interaction activities and self-esteem than their male counterparts (70). There may be some gender differences in the self-enhancement effect of old age identity. We further set the interaction terms between gender and the three indicators of old age identity to verify whether gender can moderate the influence of the three indicators of old age identity on self-esteem. The empirical results show that the positive influence degree of old age identity and aging degree on self-esteem of elderly women is significantly higher than that of men, but there is no significant gender difference in the influence of group identification of the elderly on self-esteem. Therefore, in terms of affirming the self-worth and realizing the purpose of self-enhancement, old age identity has more important significance for elderly women.

## Discussion

For RQ1, according to CLASS2014, the average old age identity of Chinese people aged 60 and above is 70 years. More than 40% of Chinese elderly people aged 60 and above don't identify with their old age status. An earlier study assessed old age identity among 666 Midwesterners in the United States, and there appeared to be a consensus across all age groups that old age begins around age 74 years (8). There is a difference of nearly 10 years in contrast to Neugarten et al. finding that old age begins around age 65 years (71). A recent study assessed 126 Israelis aged 65 and above with an average old age identity of 69 years (72). Whether it is in different periods for a country or between different countries, there are certain differences in the way that old age identities are formed; we believe this may be related to changes in individual social experiences, increased life expectancy, and differences in sociocultural backgrounds across countries.

For RQ2, this study verifies that the traditional Chinese cultural background of respect for the elderly has a significant impact on the old age identity of the Chinese elderly. After controlling for the various influencing factors, there are positive and negative effects of family factors on the old age identity of the Chinese elderly. We found that the elderly who frequently cared for grandchildren entered old age earlier, were more likely to identify with the elderly group, and experienced an exacerbated aging degree. Our conclusions differ from Kaufman and Elder, who found that the elderly who like to be grandparents feel younger, believe that old age will appear later, and that positive interactions with grandchildren may lead to younger age identity (41). We speculate that this may be related to the frequency of grandchild care, and that the elderly who care for grandchildren on a daily basis may have lower levels of physical fitness, making it difficult to demonstrate a positive old age identity. In addition, from the perspective of cultural differences between China and the West, the majority of grandparents follow a norm of “non-interference” in intergenerational relationships and do not assume a central role in caring for, and rearing grandchildren (73). However, in contemporary rural and urban China, it is increasingly common for grandparents to play a major caregiving role. Childcare provided by grandparents could be interpreted as a family adaptive strategy to maximize the wellbeing of the whole family, by alleviating the mothers' burdens and enabling them to pursue economic opportunities, and also indicates a significant cultural emphasis on collective family interests over individual interests (74). Chinese grandparents who often provide childcare may regard this as a sign of entering old age.

For RQ3, based on the social identity theory of social psychology, from the perspective of the elderly affirming their own value, we prove that in order to improve their self-esteem, the elderly tend to identify themselves as belonging to the non-elderly group, have a lower aging degree, and enter old age later. Although the theoretical connotation of old age identity also includes sociological role identity theory, Hogg et al. argue that social identity theory is more advantageous in explaining psychological mechanisms, through critical comparison of role identity theory and social identity theory (75). Theorists in the aging field have suggested that the tendency of aging adults to maintain younger subjective age identities is a form of defensive denial by which they can dissociate themselves from stigma attached to growing old (76). Recent research suggests that adults may switch from an age-group identity to a generational identity to strategically avoid negative age stereotypes and protect their positive self-views (60, 77). We argue that maintaining a positive self-view may not be a form of defensive denial for the Chinese elderly. Under the background of traditional Chinese culture of respect for the elderly, the elderly enjoy many preferential treatments, such as free bus rides, free access to parks, and free museum visits. In fact, the elderly are highly respected in China, on the contrary, those who discriminate against and disrespect the elderly are condemned by moral public opinion and even punished by law in China.

China's one-child policy, established in 1979, has had a depressive effects on fertility rates, increasing the proportion of elderly people in China and causing profound economic and social problems (78). The generation that grew up under China's one-child policy will also tend to have a lower birth rate, which will push China toward negative population growth and more serious aging population. The old age identity of the elderly in China has great practical and policy significance in coping with the challenges brought by an aging society. Based on the self-perception of the elderly on when old age begins, not all Chinese elderly people aged 60 and above identify with their old age status. The aging society may not be “aging,” which may bring forth more high-quality elderly human resources. According to CLASS2014, the average old age identity of Chinese people aged 60 and above is 70 years. In fact, delaying the retirement age may benefit the elderly who show advantages in health, psychology and socioeconomic status, and may have a certain impact on the elderly who are disadvantaged in these areas. In order to actively respond to aging, the Chinese government should pay more attention to the health and psychology of the elderly, raise the level of pension income, encourage the elderly to actively participate in politics, public welfare, etc., and strategically adjust the old age identity of the Chinese elderly. Due to the traditional Chinese cultural background of respect for the elderly, family intergenerational support can also help the elderly to show a positive old age identity; creating an elderly-friendly society will thereby, also pave an effective path for active aging.

Since the implementation of the one-child policy in China in 1979, traditional family structure and care-giving patterns in Chinese society have been changed (78), and one-child families in urban areas have become mainstream (57). The traditional Chinese culture of respect for the elderly and the norms of filial piety make the elderly, their children and grandchildren gather the strength of intergenerational solidarity and present a close intergenerational relationship. The historically hierarchical intergenerational relationship is replaced by one that emphasizes mutual care and reciprocal exchanges, reflecting a contemporary renegotiated and reinterpreted “intergenerational contract,” in which both generations make investments (79). The three-generation family structure between the elderly and their children and grandchildren enhances the age identity of the elderly through the supportive behavior of children even grandchildren to respect the elderly, however, the positive age identity that the elderly obtain also faces certain challenges. Under the four-two-one family structure (four grandparents, two parents, one child), adult children shoulder the burden of supporting both elderly parents (74), which undoubtedly brings more structural barriers to children providing support for their parents. However, other studies have shown that, although all the cultures studied share the value of elder respect, the extent to which elders are actively or passively respected, and the forms of respect shown most often would seem to vary by culture (54). Streib found that Chinese people's respect for the elderly is an automatic expression (80). Although social changes and changes in family structure may change the forms of respect for the elderly, the Chinese culture of respect for the elderly is deeply influenced by the Confucian filial piety ethics (54, 81). Values, norms, roles, and patterns of social interaction associated with respect for elders have persisted from generation to generation (53, 80). We have reason to believe that the traditional Chinese culture of respect for the elderly and the norms of filial piety will be well preserved, but the Chinese government should realize that the ability of children to perform filial piety must also be formally supported outside the family.

## Conclusions

Old age identity is the self-perception of when old age begins, reflects public age values and acceptance of aging. Paying attention to the old age identity of the elderly in China has great practical and policy significance for actively responding to the aging society. Different from Western countries, China is a country with a long-standing culture of respect for the elderly and filial piety norms, and the influence of the cultural background on the old age identity is worthy of attention. Using the 2014 China Longitudinal Aging Social Survey (CLASS), this study focuses on the old age identity of the Chinese elderly and its influencing factors. Combined with the traditional Chinese cultural background of respect for the elderly and the norms of filial piety, we build a predictive model of old age identity among the Chinese elderly, including seven dimensions: demographic correlates, socioeconomic status, health status, psychological status, behavior, major events, and family factors.

By analyzing the theoretical connotation of the concept of old age identity and using mathematical processing techniques, we obtained two other indicators based on old age identity: elderly group identity and aging degree. We found that the empirical results on the influencing factors of old age identity are also applicable to elderly group identity and aging degree. Health status, psychological status, behavior, socioeconomic status, and some demographic characteristics significantly affect the old age identity of the Chinese elderly. As common influencing factors of age identity, these findings are consistent with the conclusions of previous studies. Increasing age causes the elderly to enter old age later, raise the likelihood of identification with elderly groups, and exacerbate aging degree. Higher years of schooling, nonagricultural work, and higher pension income cause the elderly to enter old age later, raise the likelihood of identification with non-elderly groups, and reduce aging degree. Better self-assessed health, no chronic disease, higher life satisfaction status, and better emotional status also delay their entry into old age, keep them identifying with the non-old age groups, and reduce aging degree. Participating in political elections and public welfare activities also have the same positive effects on the elderly.

Cultural background factors is novel about our study, we introduce for the first time that the traditional Chinese cultural background of respect for the elderly and the norms of filial piety have an impact on the old age identity of the Chinese elderly through family intergenerational support, that haven't been addressed in previous studies. The various forms of support provided by children to the elderly can help them achieve a positive age identity. Children providing substantial financial support to the elderly, caring about them often, and helping them with housework often, enable them to enter old age later, raise the likelihood of identification with non-elderly groups, and reduce aging degree. The number of children of the elderly, the children's economic conditions and care support for grandchildren have negative effects. Larger numbers of children, financially disadvantaged children, and often caring for grandchildren cause the elderly to enter old age earlier, raise the likelihood of identification with elderly groups, and exacerbate aging degree.

In addition, based on the social identity theory of social psychology, we empirically analyzed the significance of old age identity for the elderly from the perspective of them affirming their own value. In order to maintain self-esteem, the elderly tend to identify themselves as belonging to the non-elderly group, have lower aging degree, and enter old age later. We also found that gender can moderate the influence of old age identity on self-esteem. In terms of affirming the self-worth and realizing the purpose of self-enhancement, old age identity has more important significance for elderly women.

We hope that our findings may add practical value among endeavors toward enhancing the quality of life for the elderly, worldwide.

## Limitations and future work directions

There are a few limitations of this study. First, our study is based on a sample of Chinese elderly people aged 60 and above, which may render it difficult to generalize our findings. However, the identity of different age groups may have different behavioral responses, such as their devotion to the community (82). Of course, another study points out that the age identities of middle-aged and elderly people are independent, and it is necessary to conduct separate studies (72, 83). Second, our study only involves the Chinese cultural background of respect for the elderly, emphasizing the impact of family intergenerational support on the old age identity of the Chinese elderly. However, the 5,000-year-old culture in China is extensive, profound and has a long history. Not only is the behavior of respect for the elderly rich in content, but other cultural backgrounds in China may also have an important impact on the old age identity of the Chinese elderly, this can be a future work direction. Although we are the first to conduct a more comprehensive study on the influencing factors of old age identity among the Chinese elderly using survey data, we may have also ignored some important factors, to which we hope to pay special attention, in future studies. In addition, future work directions may consider quantifying the cultural background of each country as an influencing factor of age identity and carry out comparative analysis, which will help to understand the differences of age identity among countries.

Despite these limitations, we believe that our study significantly enriches existing conclusions about age identity. We theoretically analyzed the concept of age identity and logically deduced the question of old age identity, and obtained three types of indicators. We explained the profound connotation of old age identity, which has particular significance for studies on other dimensions of age identity. Although there have been many studies on the influencing factors of age identity, they have not empirically tested the role of the cultural background, our study deepens the influence of sociocultural background on age identity. Based on the social identity theory of social psychology, we also empirically verified that the elderly achieve self-enhancement by having an old age identity, which further highlights the significance of old age identities for the elderly.

## Data availability statement

The original contributions presented in the study are included in the article/supplementary material, further inquiries can be directed to the corresponding author.

## Author contributions

XY and QW: conceptualization and methodology. QW: writing–original draft preparation. XY: writing–review and editing. All authors have read and agreed to the published version of the manuscript.
